# Upregulation of Immune checkpoint PD-L1 in Colon cancer cell lines and activation of T cells by *Leuconostoc mesenteroides*

**DOI:** 10.1007/s11274-024-04018-7

**Published:** 2024-05-17

**Authors:** Safaa Altves, Ebru Guclu, Esra Yetisgin, Kivanc Bilecen, Hasibe Vural

**Affiliations:** 1 Department of Medical Biology, Faculty of Medicine, Necmettin Erbakan University, Konya, Turkey; 2 Science and Technology Research and Application Center (BITAM), Necmettin Erbakan University, Konya, Turkey; 3 Department of Basic Science and Health, Hemp Research Institute, Yozgat Bozok University, Yozgat, Turkey; 4 Department of Molecular Biology & Genetics, Faculty of Agriculture and Natural Sciences, Konya Food and Agriculture University, Safaa ALTVES, Konya, Turkey

**Keywords:** Immunotherapy, *Leuconostoc mesenteroides*, Probiotic, PD-L1

## Abstract

**Abstract:**

Globally colorectal cancer ranks as the third most widespread disease and the third leading cause of cancer-associated mortality. Immunotherapy treatments like PD-L1 blockade have been used to inhibit the PD-L1 legend, which boosts the activity of cytotoxic T lymphocytes. Recently, studies suggest that some probiotics could potentially enhance the effectiveness of immunotherapy treatments for cancer patients. We found that in Caco-2 and HT-29 cells, the live *Leuconostoc mesenteroides* treatment resulted an increase in the PD-L1 expression and this treatment stimulated interferon-gamma (IFN-γ) production in Jurkat T-cells. Due to the well-established ability of IFN-γ to enhance PD-L1 expression, the combination of IFN-γ and *L. mesenteroides* was used in colon cancer cell lines and a resulting remarkable increase of over tenfold in PD-L1 expression was obtained. Interestingly, when *L. mesenteroides* and IFN-γ are present, the blockage of PD-L1 using PD-L1 antibodies not only improved the viability of Jurkat T-cells but also significantly boosted the levels of IFN-γ and IL-2, the T-cells activation marker cytokines. In addition to upregulating PD-L1, *L. mesenteroides* also activated Toll-like receptors (TLRs) and NOD-like receptors (NODs) pathways, specifically through TLR2 and NOD2, while also exerting a suppressive effect on autophagy in colon cancer cell lines. In conclusion, our findings demonstrate a significant upregulation of PD-L1 expression in colon cancer cells upon co-culturing with *L. mesenteroides*. Moreover, the presence of PD-L1 antibodies during co-culturing activates Jurkat T cells. The observed enhancement in PD-L1 expression may be attributed to the inhibition of the Autophagy pathway or activation of the hippo pathway.

**Graphical abstract text:**

The administration of Live *Lactobacillus mesenteroides* on colon cancer cells leads to the elevation of PD-L1, with a further increase observed in the presence of IFN-γ. Co-cultivation of Live *L. mesenteroides* with colon cancer cells in conjunction with anti-PD-L1 blockade antibody results in the enhanced viability of T cells.

**Key Points:**

- Co-culturing *L. mesenteroides* increases PD-L1 gene and protein transaction in colon cancer.
- *L. mesenteroides* existing enhances T cells viability and activity.
- GPCR41/42 is a possible link between *L. mesenteroides*, YAP-1 and PD-L1.

**Supplementary Information:**

The online version contains supplementary material available at 10.1007/s11274-024-04018-7.

## Introduction

Cancer stands as a pervasive ailment with staggering global mortality rates Annually, tens of millions of individuals grapple with a cancer diagnosis, facing an alarming 50% mortality rate (Anand et al. 2008). Among the myriad of cancers, colorectal cancer (CRC) emerges as the third-leading cause of death worldwide (Rawla et al. 2019), registering an annual incidence of over 1.2 million cases and claiming approximately 600,000 lives (Khan et al. 2014). Diverse risk factors significantly contribute to the onset of CRC. These include bacterial and viral infections, alcohol consumption, smoking, advancing age, ulcerative colitis, sedentary lifestyle, and genetic abnormalities in crucial pathways (Yaghoubi et al. 2019). Recent investigations shedding light on alterations in the gut microbiota of CRC patients suggest a potential link between gut microbiota dynamics and the genesis of CRC (Saus et al. 2019). This underscores the multifaceted nature of factors influencing colorectal cancer, ranging from genetic predispositions to environmental and lifestyle elements.

Immunotherapy represents a paradigm shift in the landscape of cancer treatment, heralding a transformative era characterized by the reinforcement of innate immune mechanisms, culminating in the elimination of malignant cells and concomitant amelioration of patient well-being. Among the diverse modalities of immunotherapeutic interventions, Immune Checkpoint Inhibitors (ICIs) stand out prominently. This therapeutic approach owes its conceptual foundation to the discernment of aberrant upregulation of programmed death-ligand 1 (PD-L1) and its receptor programmed cell death protein 1 (PD-1) across various malignancies, a phenomenon intricately linked with immune evasion mechanisms. The conceptual framework involves the use of antibodies to impede the PD-L1/PD-1 and CTLA-4 pathways. This obstruction activates T cells and augments immune surveillance. Notably, this pioneering approach was proposed by the 2018 Nobel Prize laureates, James P. Allison and Tasuku Honjo (Guo 2018). The application of this breakthrough technique has yielded success in treating diverse cancers, such as non-small cell lung cancer, melanoma, bladder cancer, and colorectal cancer (Zhang et al. 2022).

Within the mammalian gastrointestinal tract resides a vast and diverse community of commensal bacteria. The gut microbiome, having co-evolved with its host over time, confers a myriad of benefits, encompassing nutrition synthesis, digestion, protection from infections, detoxification, and modulation of the immune system (Wu and Wu 2012). The pivotal influence of the gut microbiota on the immune system has prompted investigations into its effects on the tumor microenvironment, thereby shaping tumor susceptibility to various therapeutic modalities, including immunotherapy (Ting et al. 2022).Numerous studies have underscored the substantial impact of gut microbiome composition on the efficacy of anticancer immune surveillance, particularly in the context of PD-1/PD-L1 and CTLA-4-based therapies (Guo et al. 2021). Notably, individuals with cancer undergoing immune checkpoint inhibitor (ICI) therapy, characterized by a prevalent microbiome composition of Firmicutes and Verrucomicrobia, exhibited heightened sensitivity to the treatment. Conversely, patients harboring a microbiome dominated by Proteobacteria experienced adverse treatment outcomes (Huang et al. 2021).

Human lifestyle choices can contribute to gut microbiome dysbiosis, prompting the exploration of probiotics as a method to restore microbial balance and treat dysbiosis (Wang et al. 2021). While the benefits of probiotics are multifaceted, one area necessitating thorough investigation is their potential to modulate the host immune responseAmong the lactic acid bacteria, *Leuconostoc. mesenteroides*, characterized by its gram-positive nature, coccoid-like shape, and heterofermentative properties, belongs to the Firmicutes phylum. Widely distributed in various sources such as wine, vegetables, fruits, cereals, fish, meat, and dairy products, L. mesenteroides exhibits traits of being nonmotile, non-spore-forming, anaerobic facultative, and typically appearing in pairs or short chains (Paula et al. 2015). Despite its documented positive effects, such as enhancing mucosal barriers and influencing the body’s immunological response (Matsuzaki et al. 2015), the impact of this bacterium on ICIs remains unexplored.

The principal aim of this study is to evaluate the impact of live *L. mesenteroides* on the expression levels of PD-L1gene and protein within CRC cellular models. Subsequently, we intend to scrutinize the influence of altered PD-L1 expression on the immune response by adding immune cells to the in vitro model. Additionally, our study endeavors to elucidate the potential signaling pathways implicated in the modulation of PD-L1 gene and protein expression in CRC cells.

## Materials and methods

### Isolation and identification of L. mesenteroides

*L. mesenteroides* isolation was performed using a local kefir source. To initiate the isolation, 100 mg of kefir powder was introduced into 10 ml of tryptone broth containing 10% sucrose (w/v). Subsequently, the samples were cultivated under anaerobic conditions at 25 °C for 48 h. Following incubation, 100 µl of the culture was transferred to *L. mesenteroides* (LM) broth.

LM colonies were obtained by plating on LM agar (LM broth supplemented with 12 g/L agar) following the method outlined by Dimic (2006). DNA extraction from individual colonies was performed using the conventional phenol-chloroform technique, as detailed by Bilecen et al. (2015). The amplification of the 16S rRNA gene was carried out through PCR, employing universal primers 008F (5’-AGAGTTTGATCMTGGC-3’) and 1387R (5′-GGGCGGWGTGTACAAGRC-3’) as described by Klindworth et al. (2013). The PCR conditions included an initial denaturation step of 10 min at 95 °C, followed by 35 cycles comprising 45 s at 95 °C, 45 s at 57 °C, and 1 min and 15 s at 72 °C. The resulting DNA fragments were subsequently sequenced by 3130 Genetic Analyzer and analyzed.

## Tolerance to pH and bile salt

In a concise procedure, 50 µl of an overnight culture was introduced to 950 µl of LM media containing varying concentrations of bile salt (0.3% or 0.5%, w/v) or adjusted to different pH values (2, 3, and 4). Subsequent to incubation at 37 °C for 3 h, pH and bile salt tolerance were evaluated by enumerating viable cells on LM agar plates. The assessment was conducted utilizing the formula

$$\text{S}\text{u}\text{r}\text{v}\text{i}\text{v}\text{a}\text{l} \text{r}\text{a}\text{t}\text{e} \left(\text{\%}\right)=\frac{\# \text{o}\text{f} \text{f}\text{i}\text{n}\text{a}\text{l} \left(\text{c}\text{f}\text{u}\right)}{\# \text{o}\text{f} \text{i}\text{n}\text{i}\text{t}\text{i}\text{a}\text{l} \left(\text{c}\text{f}\text{u}\right)}\times 100$$

**Adhesion of*L. mesenteroides*to colon cells**.

To assess the adhesive capabilities of *L. mesenteroides*, Caco-2 and HT-29 cells were seeded into 96-well microplates at a density of 4,000 cells per well. Subsequently, the cells were incubated at 37 °C with 5% CO_2_ for 24 h. Following the incubation period, the cells were exposed to *L. mesenteroides* for 2 h. Post-treatment, the cells underwent three washes with sterile PBS, and 5% Triton X-100 was then added to lyse the cells. The resulting lysates were plated onto LM agar plates in varying dilutions to facilitate the enumeration of adherent bacteria. The final adhesion rate was determined using the following equation (Letourneau et al., 2011):

$$\text{a}\text{d}\text{h}\text{e}\text{r}\text{e}\text{d} \text{b}\text{a}\text{c}\text{t}\text{e}\text{r}\text{i}\text{a} \left(\text{\%}\right)=\frac{\# \text{o}\text{f} \text{a}\text{d}\text{h}\text{e}\text{r}\text{e}\text{d} \text{b}\text{a}\text{c}\text{t}\text{e}\text{r}\text{i}\text{a} \left(\text{c}\text{f}\text{u}\right)}{\# \text{o}\text{f} \text{i}\text{n}\text{i}\text{t}\text{i}\text{a}\text{l} \text{i}\text{n}\text{o}\text{c}\text{u}\text{l}\text{u}\text{m} \left(\text{c}\text{f}\text{u}\right)}\times 100$$

### Antibiotic susceptibility

The susceptibility of *L. mesenteroides* to various antibiotics (gentamicin, CN; ampicillin, AMP; vancomycin, VA; erythromycin, E; ciprofloxacin, CIP; cefoxitin, FOX) was examined by the conventional disc diffusion method.

## Hemolysis test

*L. mesenteroides* was cultured on blood agar plates (blood agar base, Himedia #M073-100G with 5% sheep blood) then incubated at 30^ο^C for 24 h.

### Preparation of bacterial cultures for co-culturing with CRC cells

Bacteria were cultivated in LM broth at 30 °C without agitation. Subsequent to incubation, bacteria were harvested during the late-log/early stationary phase, corresponding to a concentration of 10^7^ CFU/mL. After centrifugation at 4,500 rpm for 10 min at 4 °C, the bacterial pellet was washed with sterile PBS (pH 7.2). The bacterial suspension was then adjusted to the desired colony numbers (10^6^-10^9^ CFU/Cell) using antibiotic-free high-glucose Dulbecco’s Modified Eagle Medium (hDMEM). Enumeration of the total number of bacteria (CFU/mL) was determined through serial dilution methods. Additionally, the relationship between bacterial optical density at 600 nm (OD_600_) and CFU/mL was calculated.

### Cell culture

To investigate the impact of *L. mesenteroides* on PD-L1 expression, we selected two CRC cell lines: Caco-2 (ATCC^®^ HTB-37™) and HT-29 (ATCC^®^ HTB-38™). This choice was motivated by the understanding that cancer initiation often involves alterations in specific cell types. By utilizing two distinct CRC cell lines, we aimed to discern any differential responses to *L. mesenteroides* co-culturing, thus providing insights into potential variations in PD-L1 expression dynamics across CRC contexts. Furthermore, to mimic the in vivo immune response of colon cells to *L. mesenteroides*, we utilized Jurkat T cell E6-1 clone (ATCC^®^ TIB-152™) as immune cell. All cells were procured from the American Type Culture Collection. CRC cells lines Caco-2 and HT-29 were cultured in hDMEM, supplemented with 10% fetal bovine serum (FBS) and penicillin-streptomycin (10 U/mL and 100 mg/mL, respectively). Cell detachment, upon reaching 80% confluence, was achieved using trypsin-EDTA. Jurkat T cells were cultured in Roswell Park Memorial Institute Medium (RPMI) supplemented with L-glutamine, antibiotics, and 5% fetal bovine serum (FBS). Mimicking the two-signal activation process required for T cell activation in vivo, we utilized the ImmunoCult™ Human CD3/CD28 T Cell Activator (STEMCELL, #10,971) according to the manufacturer’s protocol. This approach ensured that pre-activated Jurkat T cells were used in immune response experiments. All cell lines were maintained at 37 °C in a 5% CO_2_ environment.

### Cell viability analysis

To study the effect of *L. mesenteroides* on human colon cancer cell line XTT assay from Biological Industries (Catalog #20-300-100) was used. In brief, 100 µL of cells were seeded into 96-well microplates at a concentration of 2,000 cells/well and incubated for 24 h at 37 °C with 5% CO_2_. Subsequently, various concentrations of live bacteria (10^6^, 10^7^, 10^8^, and 10^9^ cfu) were introduced into each well, followed by further incubation under the same conditions. Following the incubation period, the medium was aspirated and replaced with XTT reagent, following the manufacturer’s protocol. Untreated colon cancer cells served as controls.

### Real-time PCR

Gene expression levels were quantified through reverse transcription quantitative polymerase chain reaction (RT-qPCR). Total RNA extraction from colon cancer cells was performed using Trizol RiboEx (GeneAll, Catalog #301-001). Subsequently, the RNA samples underwent DNAse I treatment (Thermo Scientific, Catalog #EN0521). Reverse transcription from RNA to cDNA was achieved using the iScriptTM cDNA Synthesis Kit (Bio-Rad, Catalog #170–8891). The subsequent qPCR was carried out with BrightGreen master mix (BrightGreen qPCR MasterMixR) and 0.5 pmol of each primer. The qPCR conditions were set as follows: initial denaturation for 10 min at 95 °C, followed by 40 cycles of denaturation at 95 °C for 15 s and annealing/extension at 60 °C for 60 s. Real-time data analysis was conducted using the 2^–ΔΔCT^ method, and the transcription levels of the targeted genes were normalized to housekeeping genes actin beta (ACTB) and glyceraldehyde-3-phosphate dehydrogenase (GAPDH) reference genes.

### Western blot analysis

Changes in the PD-L1 protein level were investigated through Western blot analysis,. In summary, total proteins from colon cancer cells were separated using SDS-PAGE and subsequently transferred to a PVDF membrane using the Trans-Blot Turbo Transfer System (BioRad) at 25 V for 7 min. Blocking of the membranes was performed using 3% skimmed milk. The membranes were then incubated overnight with primary antibodies against PD-L1 (Bioss, Catalog #BS10159R, 1:1000) and β-Actin (Bioss Antibodies, Catalog #BS-0061R, 1:1000) at 4 °C. Following three washes with TBST, secondary antibodies (Jackson Immuno Research, Catalog #211-035-109, 1:10,000) were applied. The bands were visualized using a ChemiDocTM MP imaging system (BioRad) with a chemiluminescent enhancer substrate (Elabscience^®^ excellent chemiluminescent substrate Kit, Catalog #E-IR-R301). Image Lab^®^ software (BioRad) was employed for the analysis of band density.

### Mimicking the in vivo conditions

We hypothesis that increasing of PD-L1 gene and protein expression in colon cancer cells may affect the immune response and to investigate this observation. We designed a new experiment similar to (Sahler et al. 2018). Colon cancer cells Caco-2 or HT-29 cells were seeded in 6-well plates at a concentration of 20,000 cells/well and incubated for 24 h at 37 °C with 5% CO_2_. Subsequently, 1 × 10^6^ live bacteria and IFN-γ were added to each well and further incubated for 6 hours for Caco-2 and 24 h for HT-29 at 37 °C in a 5% CO_2_ incubator. Following the incubation, the medium was aspirated, and cells were washed three times with PBS. A new complete RPMI media, supplemented with 50 µg/ml gentamicin (Sigma #058M4859V), 0.05 µg/ml PD-L1 antibody (Elabscience^®^, Anti-Human CD274 Antibody, #E-AB-F11330), and 200,000 pre-activated Jurkat T cells, were added in a 1:10 (cell/T-cell) infectious ratio. These co-cultures were further incubated for 24 h at 37 °C in a 5% CO_2_ incubator. Finally, the supernatants were collected to measure T cells cytokines (IL-2 and IFN-γ) and to evaluate T cells viability.

### ELISA assays

IL-2 and IFN-γ levels in T cells were quantified using ELISA assays from BT LAB (Catalog #E009Hu for IL-2 and #E0105Hu for IFN-γ). In summary, 10 µl of anti-IL-2 or anti-IFN-γ was added to 40 µl of supernatant in 96-well plates. Each sample was then supplemented with 50 µl of streptavidin-HRP. The samples were covered and incubated for one hour at 37 °C, followed by five washes with washing buffer.Subsequently, 50 µL of substrate solution A and 50 µL of substrate solution B were added to each well, and the samples were re-incubated for 10 min at 37 °C in subdued lighting conditions. Finally, 50 µL of stop solution was added to each well, and the yellow color developed was measured at 450 nm within 10 min.

### Assessment of T-cells viability

The determination of T cells’ viability was conducted using the FITC Annexin V apoptosis kit (BioLegend, Catalog #640,922), following the procedures outlined in the accompanying data sheet. The analysis was performed utilizing a flow cytometry device, specifically the BD FACSAriaTM III.

### Statistical analysis

Each experiment was conducted in triplicate, and the results were presented as the mean ± standard deviation. Statistical analyses, including the Student’s t-test, one-way ANOVA, and two-way ANOVA, were performed using GraphPad Prism 5^®^ to assess the data. A significance threshold of *p* < 0.05 was applied to determine statistical significance.

## Result

### Identification and characterization of *L. mesenteroides*

**Fig. 1:**
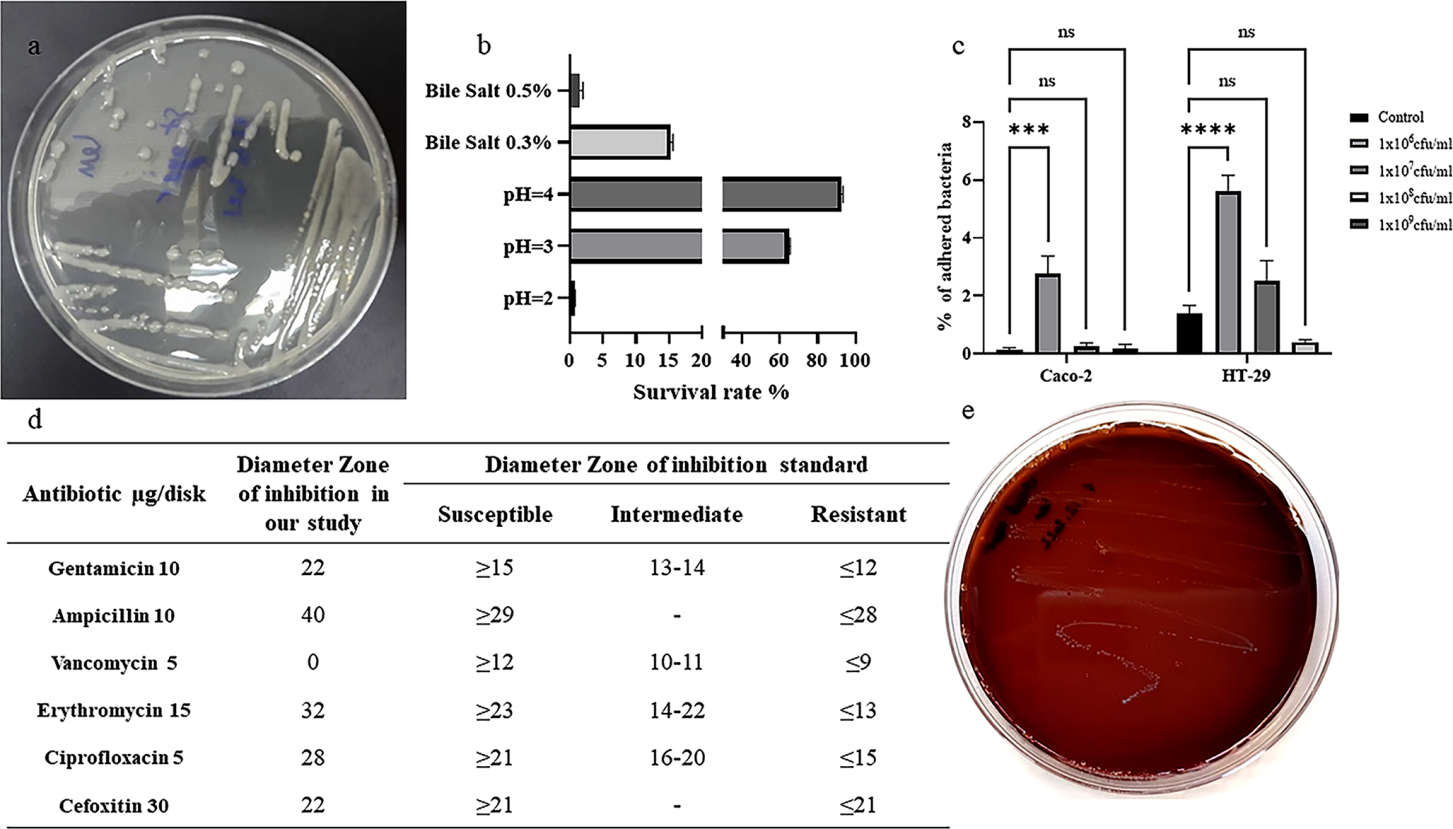
*Leuconostoc mesenteroides* strain isolated from the kefir sample. **(a) ***L. mesenteroides* colonies on LM agar plate (37℃, 5% CO_2_, 24 h); **(b)** Tolerance of *L. mesenteroides* to low pH (2, 3, 4) and bile salt (0.3%, 0.5%); **(c)** Adhesion of *L. mesenteroides* to Caco-2 and HT-29 cells (given as percent bacteria adhered); **(d)** Antibiotic susceptibility of *L. mesenteroides* to various antibiotics: vancomycin, gentamicin, ciprofloxacin, erythromycin, ampicillin, cefoxitin; **(e)** Hemolysis test of *L. mesenteroides* on blood agar plate. Data were analyzed with GraphPad Prism using one way ANOVA test p < 0.05 (*)

*L. mesenteroides* was isolated from kefir using a selective medium. It produced dextran, giving colonies a slimy appearance. Identified as Gram-positive cocci, it lacked catalase and oxidase activities. Two methods confirmed its identity: “16S-based ID” at ezbiocloud.net and BLAST at NCBI, matching *L. mesenteroides* subsp. mesenteroides ATCC 8293 and *L. mesenteroides* subp. Dextranicum DSM 20,484. Phylogenetic analysis placed it close to these subspecies. Its DNA sequence was deposited in NCBI as “*Leuconostoc mesenteroides* subsp. mesenteroides Kefir” (accession number SUB12874666). Additionally, the isolated strain, denoted as PSC107, was deposited at the Food Engineering Department at Pamukkale University (PUFECC, WDCM 1019) phylogenetic tree in supplemental material. The study investigated various probiotic characteristics of *L. mesenteroides*. Firstly, its tolerance to acidity and bile salts was examined, showing survival rates at different pH levels and bile salt concentrations. Secondly, adhesion to Caco-2 and HT-29 cell lines was observed, though neither showed significant levels. Thirdly, antibiotic susceptibility revealed resistance to vancomycin and susceptibility to other antibiotics. Finally, the hemolysis test indicated γ-hemolytic properties, with no observed hemolysis (see Fig. 1).

### The effect of co-cultivation of *L. mesenteroides* on colon cancer cells

**Fig. 2:**
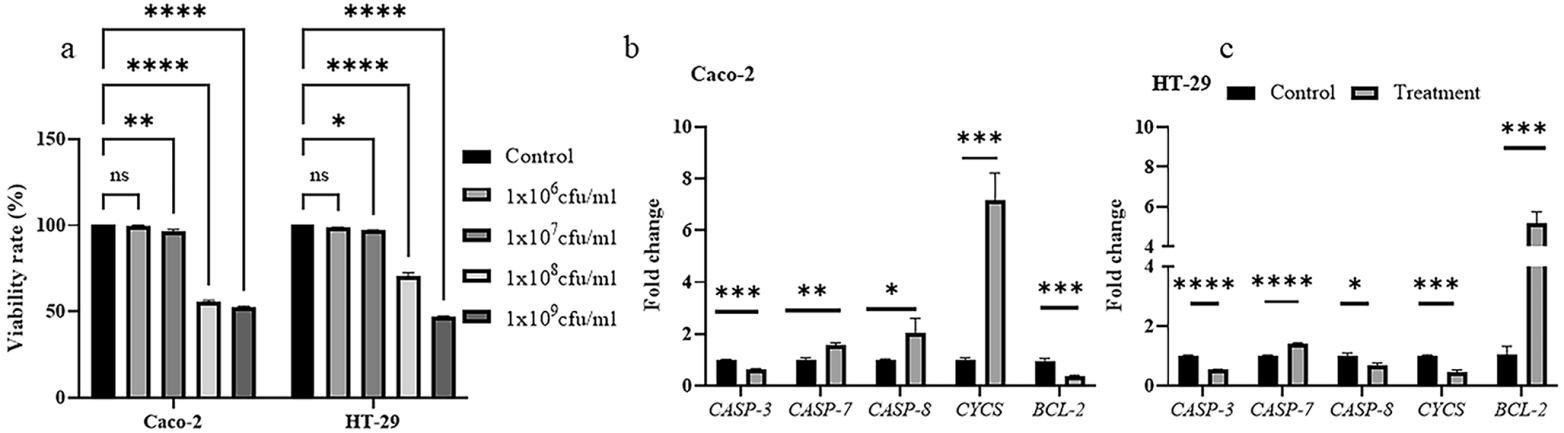
Effect of *L. mesenteroides* co-culturing on the viability of Caco-2 and HT-29 cells and changes in transcription levels of apoptosis-related genes. **(a)** Percent viability of Caco-2 and HT-29 cells in response to co-culturing (37℃, 5% CO_2_, 24 h) with various amounts of *L. mesenteroides* bacterial cells (10^6^, 10^7^, 10^8^, 10^9^ cfu/ml); **(b)**, **(c)** Effect of *L. mesenteroides* co-culturing on the transcriptional levels of selected apoptotic marker genes. *Data were analyzed with GraphPad Prism using one way ANOVA and multiple T test.p* < 0.05 (*), *p* < 0.001 (**), *p* < 0.0001 (***), *p* < 0.00001 (****), and not significant (ns) when *p > 0.05*

The study evaluated the impact of *L. mesenteroides* on colon cancer cells’ viability and apoptotic marker transcription (caspase 3 (*CASP3*), caspase 7 (*CASP7*), caspase 8 (*CASP8*), cytochrome c (*CYCS*), BCL-2 apoptosis regulator (*BCL-2*)), as well as its influence on PD-L1 transcription and protein levels. Results showed that lower doses of *L. mesenteroides* did not induce cell death but higher doses did (see Fig. 2). Transcription levels of apoptotic markers varied in response to *L. mesenteroides* in CRC cells, for example *CASP3* decreased and *CASP7* increased in both cell lines, while *CASP8* and *CYCS* increased in Caco-2 but decreased in HT-29 cells. Notably, antiapoptotic marker *BCL*-2 transcription decreased in Caco-2 but increased in HT-29 cells.

**Fig. 3:**
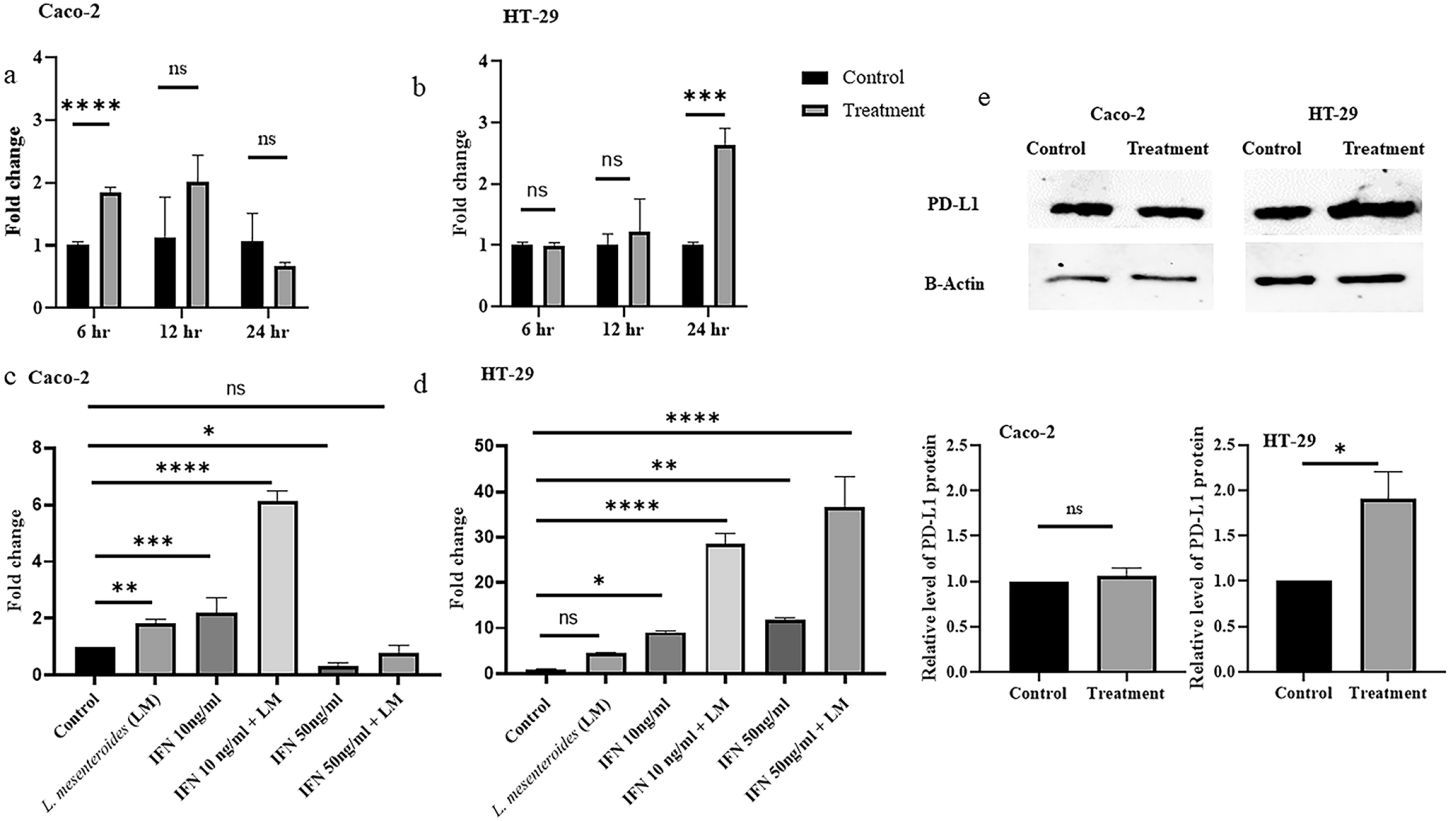
Effect of *L. mesenteroides* co-culturing on PD-L1 expression levels. **(a), (b)** Relative transcriptional levels of *PD-L1* in Caco-2 and HT-29 cells co-cultured with *L. mesenteroides* at different incubation time points (6 h, 12 h, and 24 h co-culturing at 37℃, 5% CO_2_); **(c), (d)** Relative transcriptional levels of *PD-L1* in Caco-2 and HT-29 cells with or without *L. mesenteroides* co-culturing in the presence of 10 or 50 ng/ml IFN-γ. **(e)** Relative PD-L1 protein levels in Caco-2 and HT-29 cells with or without *L. mesenteroides* co-culturing in the presence of 10 and 50 ng/ml IFN-γ. β-actin used as internal loading control. *Data were analyzed with GraphPad Prism using multiple T test, one way ANOVA, and T testp* < 0.05 (*), *p* < 0.001 (**), *p* < 0.0001 (***), *p* < 0.00001 (****), and not significant (ns) *p > 0.05*

Co-culturing with *L. mesenteroides* notably boosted PD-L1 transcription in both cell lines, particularly when combined with IFN-γ. The addition of IFN-γ aimed to mimic in vivo conditions, as previous research has demonstrated that IFN-γ increases PD-L1 gene expression in tumor tissues. Additionally, in vivo studies have shown that probiotic bacteria can stimulate immune cells to produce IFN-γ, further supporting the relevance of this experimental setup to real-world conditions. In protein-level analysis, PD-L1 expression increased in both CRC cell lines. Although the rise in PD-L1 levels was not statistically significant in Caco-2 cells, it reached a significant 1.9-fold increase over the control in HT-29 cells (see Fig. 3).

### Effect of increased PD-L1 by *L. mesenteroides* induction on immune response

**Fig. 4:**
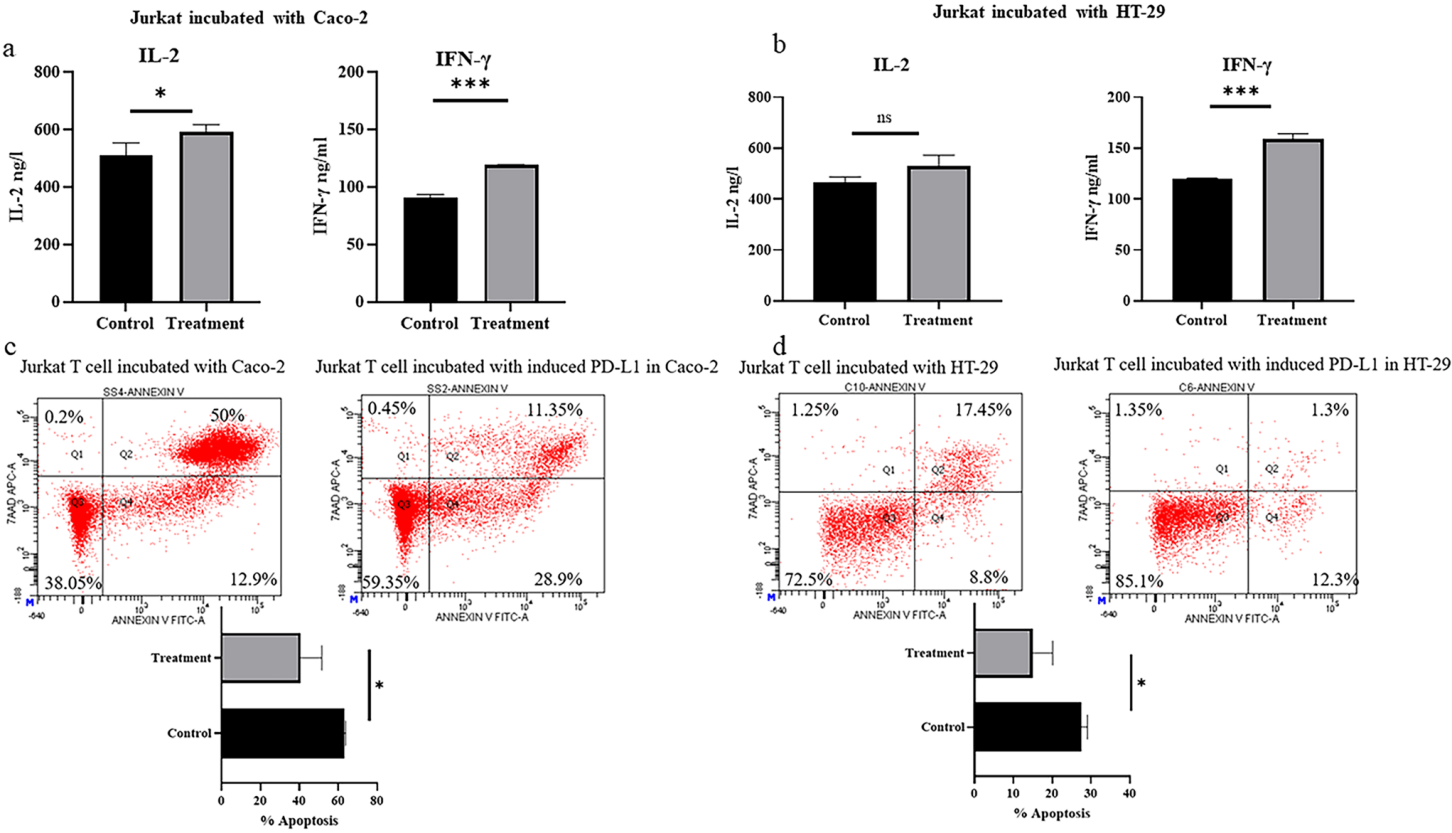
Functional analysis of pre-treated colon cancer with *L. mesenteroides* on T cell activation. **(a), (b)** IFN-γ and IL-2 levels in Jurkat T cells co-cultured with pre-treated Caco-2 and HT-29 cells with *L. mesenteroides* measured via ELISA; **(c), (d)** The viability of Jurkat T cells co-cultured with pre-treated Caco-2 and HT-29 cells with *L. mesenteroides* evaluated via FITC Annexin V apoptosis assay. *Data were analyzed with GraphPad Prism using T test p* < 0.05 (*), *p* < 0.0001 (***), and not significant (ns) *p > 0.05*

Based on previous findings, we sought to examine the impact of increased PD-L1 on immune response. To investigate this, Jurkat T cells were introduced as immune cells into the experiment, as detailed in the methods section. Analysis of cytokine levels and viability of Jurkat T cells revealed a substantial increase in IFN-γ and IL-2 levels when co-cultured with Caco-2 cells pre-treated with *L. mesenteroides*. Similarly, there was a significant elevation in IFN-γ levels in Jurkat T cells co-cultured with pre-treated HT-29 cells compared to the control group (refer to Fig. 4, A & B). Furthermore, assessment of Jurkat T cell viability using the FITC Annexin V apoptosis assay demonstrated a marked decrease in apoptosis rate in Jurkat T cells co-cultured with pre-treated colon cancer cells with *L. mesenteroides* (refer to Fig. 4, C & D).

### Relationship between *L. mesenteroides*-induced PD-L1 and bacteria-related immune pathways in colon cancer cells

**Fig. 5:**
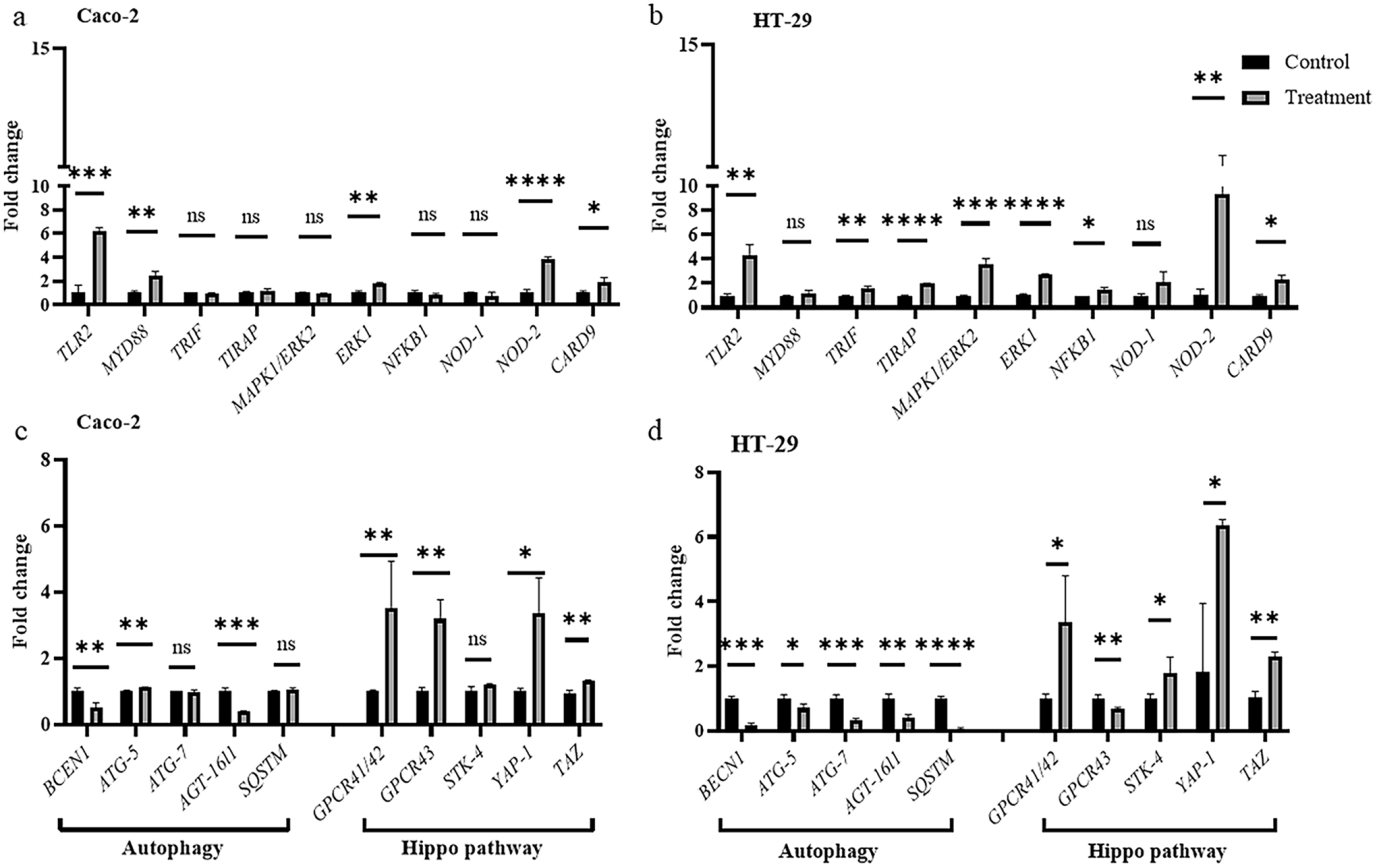
The activation of TLRs/NODs pathways and evaluation of autophagy and Hippo pathway in Caco-2 and HT-29 cells. **(a), (b)** Relative transcription levels of selected target genes in TLRs and NODs pathways in Caco-2 and HT-29 cells co-cultured with *L. mesenteroides* in the presence of IFN-γ; **(c), (d)** Relative transcription levels of selected target genes in autophagy and Hippo pathways in Caco-2 and HT-29 cells co-cultured with *L. mesenteroides* in the presence of IFN-γ. *Data were analyzed with GraphPad Prism using multiple T test p* < 0.05 (*), *p* < 0.001 (**), *p* < 0.0001 (***), *p* < 0.00001 (****), and not significant (ns) *p > 0.05*

The heightened in PD-L1 expression in CRC prompted an investigation into the pathways activated by *L. mesenteroides*. Typically, bacteria initiate cellular responses through Toll-like receptors (TLRs) and Nucleotide-binding oligomerization domain (NOD)-like receptors (NLRs), acting as sensors for bacterial contact. Our study found a significant increasing in transcriptional level of TLR2 and NOD2 along with their downstream genes, in both cell lines. This culminated in the upregulation of transcription factors such as extracellular signal-regulated kinases 1 and 2 (ERK1), ERK2, and nuclear factor kappa B Subunit 1 (NFKB1), known to regulate PD-L1 expression (refer to Fig. 5, A& B).

**Fig. 6:**
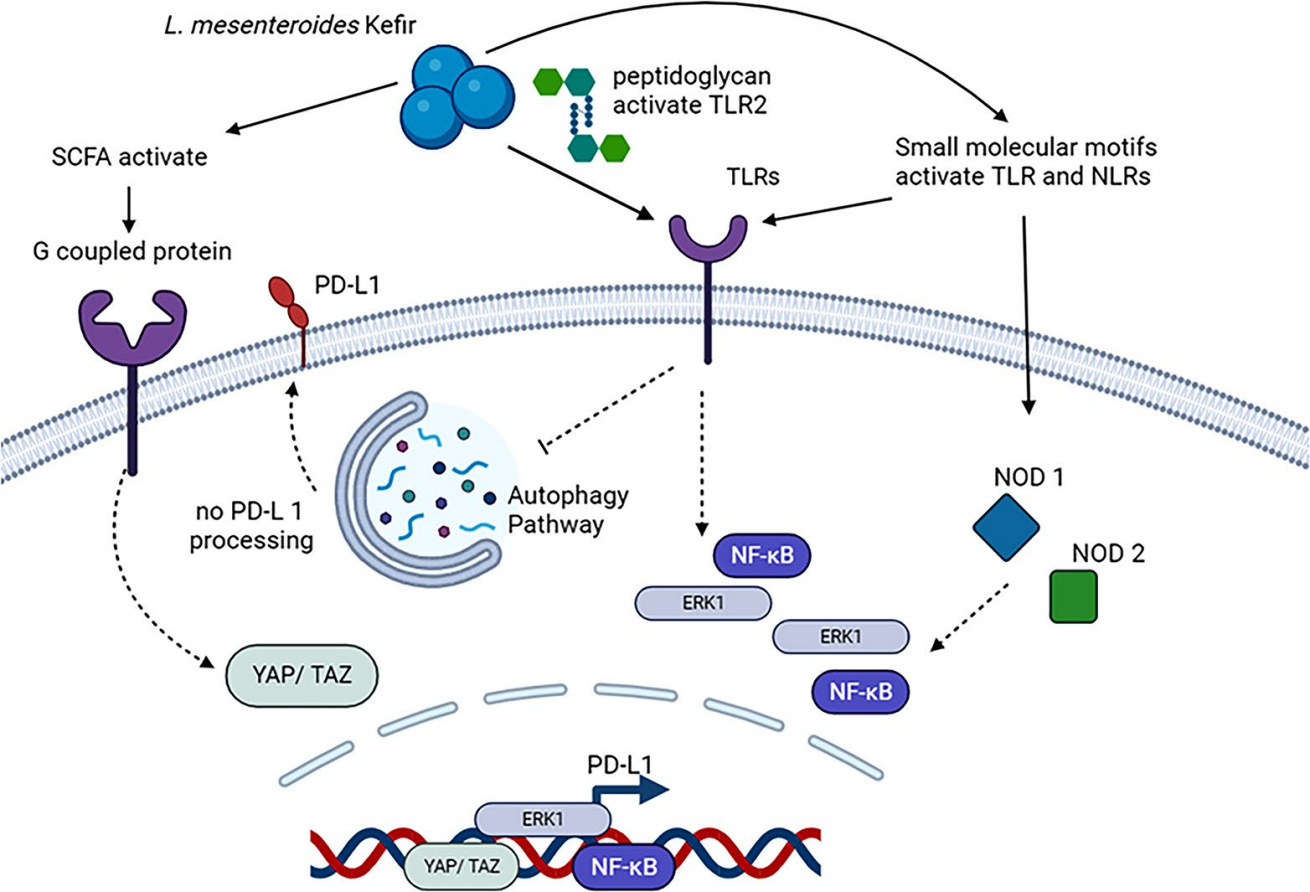
The potential cellular pathways through which live *L. mesenteroides* may upregulate PD-L1. The upregulation of PD-L1 involves the activation of TLRs, notably TLR2, and NLRs. The presence of peptidoglycan, a constituent of live *L. mesenteroides*, can activate these receptors, initiating signaling cascades that ultimately result in increased PD-L1 expression. These sensors may also impact autophagy, as evidenced by its inhibition during live *L. mesenteroides* treatment. This inhibition could, in turn, reduce PD-L1 degradation in phagosomes, contributing to the sustained expression of PD-L1 on the cell surface. Additionally, the activation of GPCRs, specifically GPR41/42 and GPR43, by SCFAs produced by *L. mesenteroides* could play a role in PD-L1 upregulation by suppressing hippo pathway thus dephosphorylated YAP and TAZ allowing them to translocated into the nucleus which may upregulate PD-L1. The interplay between these pathways may contribute to the modulation of PD-L1 gene and protein expression in response to live *L. mesenteroides* exposure

Autophagy is other mechanism in cell has a connection between bacterial infection and PD-L1 expression our results found that all.

autophagy marker genes in HT-29 cells autophagy related 5 (*ATG-5*), autophagy related 7 (*ATG-7*), beclin 1 (*BECN1*), autophagy related 16 like 1(*ATG-*16L1), Sequestosome 1(*SQSTM*) reduced significantly transcriptional activity. However, in Caco-2 just *BECN1* and *ATG-*16L1 were significantly decreased (Fig. 5). Recent research indicates that the Hippo pathway plays a crucial role in regulating PD-L1 expression, potentially connecting pathway activation with bacterial metabolism. In this study, the expression of specific genes related to the Hippo pathway (such as Serine/threonine-protein kinase 4 (*STK-4)*, Yes-associated nucleoprotein 1 gene (*YAP-1*), and transcriptional coactivator with PDZ-binding motif (*TAZ*)) and two mammalian G protein-coupled receptor genes (GPCR41/42 and GPCR43) were examined. Upon induction of PD-L1 through *L. mesenteroides* co-culturing, transcription levels of these genes increased in both CRC cells. These findings suggest a complex interplay between the Hippo pathway, GPCR genes, and PD-L1 expression in response to bacterial co-culture (Figs. 5 and 6).

## Discussion

Recent research has highlighted the importance of a healthy microbiome in improving the efficacy of immunotherapy by enhancing immune surveillance. Immunotherapeutic agents such as anti-PD-L1 antibodies function by blocking PD-L1, thereby increasing the effectiveness of cytotoxic T cells against tumors. Overexpression of PD-L1, the ligand for PD-1, is associated with decreased cytotoxic T cell activity, elevated tumorigenesis, and enhanced tumor invasiveness, all of which can be countered by anti-PD-L1 monoclonal antibody therapy (Shui et al. 2020). In our investigation, *L. mesenteroides* was isolated from a local kefir product and identified as *L. mesenteroides* subp. mesenteroides Kefir based on 16 S rRNA sequencing. This bacterium exhibited characteristic traits such as the formation of slimy colonies on sucrose-containing media due to dextran production and a lack of catalase and cytochrome c activity. Probiotic strains like *L. mesenteroides* Kefir must meet specific criteria to colonize the gastrointestinal tract effectively, including surviving stomach acidity and bile salt exposure, adhering to intestinal cells, and being non-pathogenic. Our findings suggest that *L. mesenteroides* Kefir possesses potential probiotic properties due to its ability to withstand low pH and bile salt exposure while lacking pathogenic characteristics. However, it’s important to note that the in vitro tolerance of *L. mesenteroides* Kefir to bile acids may differ from in vivo conditions influenced by factors like meal composition, and pancreatic enzyme output, and gut microbiome composition. Further research is needed to fully understand the probiotic potential of *L. mesenteroides* Kefir in vivo (Urdaneta and Casadesús 2017). High concentrations of probiotics in dietary supplements raise concerns regarding the potential transfer of antibiotic-resistant genes to intestinal pathogens, highlighting the importance of cautious use (Zheng et al. 2017). Our findings indicate that *L. mesenteroides* Kefir lacks resistance to most antibiotics except vancomycin, commonly used in selective media for its isolation (Benkerroum et al. 1993). In the context of pathogenic bacteria, hemolysis is a well-known virulence factor. Assessment of the hemolytic potential of *L. mesenteroides* Kefir on blood agar plates did not reveal any hemolysis. This finding aligns with a previous study indicating the absence of hemolytic activity in kefir-derived *L. mesenteroides* (Chang-Liao et al. 2020). Despite its potential benefits, it’s crucial to note that probiotics can vary in their adherence to intestinal cells, which could influence their ability to compete with pathogens for binding sites (Monteagudo-Mera et al. 2019). Our investigation found that *L. mesenteroides* Kefir exhibited low adherence to colon cancer cells in vitro, suggesting potential limitations in its ability to colonize the intestine and compete with pathogens. The process of bacterial adherence to mammalian cells is multifactorial, involving various factors such as bacterial pili, surface components of both bacteria and mammals including carbohydrates, proteins, and lipids (Martino 2018).

In our study, XTT analyses revealed that incubating live *L. mesenteroides* with colon cancer cells for 24 h resulted in dose-dependent cytotoxicity. The probiotic effect is influenced by various factors including dose concentrations, incubation time, and strain characteristics. While a dose of 1 × 10^6^ cfu of *L. mesenteroides* Kefir did not exhibit significant cytotoxicity in Caco-2 cells, qPCR results showed a significant increase in apoptotic marker genes. Conversely, a 6-hour co-incubation of *L. mesenteroides* Kefir induced *PD-L1* gene expression and reduced apoptotic marker genes in Caco-2 cells. In HT-29 cells, co-incubation significantly induced *PD-L1* expression after 24 h, along with an increase in anti-apoptotic *BCL*-2 gene expression. Lee et al. (2021) reported similar findings where the mRNA and protein levels of PD-L1 decreased due to the activation of *CASP-3* and *CASP-7* in HT-29 cells treated with *E. coli* K12 cells compared to the control. The differences observed between the two colon cancer cell lines can be attributed to their distinct physiological characteristics. Caco-2 cells resemble intestinal epithelial cells, while HT-29 cells are primarily undifferentiated and heterogeneous (Gagnon et al. 2013). Consistent with our observations, Süloğlu et al. (2019) demonstrated that Caco-2 cells exhibited a faster apoptotic response to Hypericin mediated photodynamic therapy compared to HT-29 cells, underscoring the differences in cellular physiology.

The knowledge that certain probiotic bacteria can stimulate T cells to produce IFN-γ is well-documented (Kekkonen et al. 2008). In our investigation, we observed that *L. mesenteroides* Kefir induced the production of IFN-γ from Jurkat T cells (data not shown). Considering that the upregulation of PD-L1 is influenced by IFN-γ, we introduced external IFN-γ to our experimental conditions. Consequently, we noted a significant increase in the mRNA level of *PD-L1* when *L. mesenteroides* Kefir was combined with IFN-γ in both cell lines compared to control. The optimal dose of IFN-γ for Caco-2 cells was determined to be 10 ng/ml, while 50 ng/ml significantly reduced PD-L1 expression compared to the control. This reduction might be attributed to induced cell death, as several studies have illustrated a dose-response relationship between IFN-γ and cell death (Rakshit et al. 2014).

Our investigation revealed distinct immunomodulatory effects contingent upon the co-culturing of colon cancer cells with pre-activated Jurkat T cells. This interaction precipitated apoptosis and concurrently diminished INF-γ and IL-2 secretions in Jurkat T cells. Intriguingly, the converse scenario, involving the pre-treatment of colon cancer cells with *L. mesenteroides* Kefir in conjunction with anti-PD-L1 blockade, yielded contrasting outcomes. Notably, this condition attenuated apoptosis and substantially augmented the secretion of both INF-γ and IL-2 from Jurkat T cells. These discerned outcomes underscore the prospective immunoregulatory role of *L. mesenteroides* Kefir, particularly in bolstering T cell stability within the context of colon cancer. Furthermore, these findings advocate for the potential utility of *L. mesenteroides* Kefir as an adjunct therapeutic agent in tandem with PD-L1 blockade interventions in vivo. In consonance with our findings, Wang et al. (2018) demonstrated analogous phenomena in the context of bladder cancer, wherein *Bacillus Calmette*–*Guérin* instigated PD-L1 gene expression. Their proposition of a synergistic interplay between bacteria and anti-PD-L1 blockades in orchestrating an immune response against bladder cancer sheds light on potential avenues for therapeutic exploration. Parallelly, Gao et al. (2021) reported analogous trends for *Fusobacterium nucleatum* in colorectal cancer. The demonstrated ability of this bacterium to enhance PD-L1 expression, amplify concentrations of INF-γ and cytotoxic T cell and augment tumor sensitivity to PD-L1 blockade suggests intriguing prospects for future therapeutic interventions.

During our research we wanted to discover the pathways that activated during *L. mesenteroides* Kefir co-culturing with human cells. And our results showed activation of TLRs, NLRs and hippo pathway (YAP/TAZ) while inhibit autophagy. TLRs and NLRs are represent pivotal components of the innate immune system, orchestrating rapid responses to pathogenic incursions or tissue damage. The activation of these receptors not only prompts the recruitment of innate immune cells but also sets in motion the activation of the adaptive immune system, as elucidated by Fukata et al. (2009). Our findings unveiled that the co-administration of *L. mesenteroides* Kefir with IFN-γ induced the activation of TLRs and NODs pathways across both cell lines. This activation, in a cascading manner, further triggered the activation of ERK1/2 and NF-κB, culminating in the upregulation of PD-L1. Numerous studies have independently corroborated the association between the expressions of ERK1, NF-κB, and the heightened expression of PD-L1 (Liu et al. 2022; Antonangeli et al. 2020; Jiang et al. 2019).

Autophagy is an orchestrated process to collect damaged or aging organelles, misfolded or mutant proteins, and other abnormalities into an autophagosome – a double-membrane vesicle, in the cytoplasm. Autophagosomes and lysosomes fusions generate autolysosomes. The components of which can subsequently be employed to breakdown the autolysosomes’ contents (Gao & Chen, 2021). In our study we found that autophagy marker genes were significantly reduced in colon cancer cell lines in response to co-culturing with *L. mesenteroides* Kefir. Similarly, in Huang et al. (2019) study, they found that the transcription of PD-L1 in the neutrophils that in lungs boosted dramatically. Also, it had been found that PD-L1 induced by Lipopolysaccharides (LPS) can be inhibited by autophagy agonists, and promoted by autophagy inhibitors which infer to the direct relationship between autophagy and PD-L1 expression.

The Hippo pathway is a crucial signaling pathway in cell biology that regulates tissue growth, organ size, and cell fate. It plays a fundamental role in maintaining tissue homeostasis by controlling cell proliferation, apoptosis, and stem cell self-renewal. The core components of the Hippo pathway include a cascade of protein kinases and transcriptional coactivators. When the pathway is active, the kinase cascade phosphorylates and inhibits the transcriptional coactivators YAP and TAZ. As a result, YAP and TAZ are retained in the cytoplasm and prevented from entering the nucleus to activate target genes involved in cell proliferation and survival. Conversely, when the Hippo pathway is suppressed, YAP and TAZ are dephosphorylated and translocated into the nucleus, where they bind to transcription factors and stimulate the expression of genes that promote cell growth and proliferation (Misra and Irvine 2018). Recent research has associated Hippo pathway with heightened PD-L1 expression. Our study explores the link between *L. mesenteroides* Kefir, PD-L1 activation, and the Hippo pathway, particularly focusing on GPR41/42 and GPR43, known to interact with short-chain fatty acids (SCFA). Co-culturing with *L. mesenteroides* Kefir led to increased expression of GPCR43 and GPCR41/42, alongside upregulated Hippo pathway marker genes, possibly contributing to PD-L1 upregulation. It is kwon from (Yu et al. 2012) that activation GPCR suppress Hippo pathway thus dephosphorylated YAP and TAZ allowing them to translocated into the nucleus which may upregulate PD-L1. Moreover, *YAP-1* overexpression in our study may suppress autophagy in colon cancer cells through elevated *BCL*-2 levels since YAP and TEAD cooperation promotes *BCL*-2 production, potentially enhancing cell survival by deterring autophagy-related cell death (see Fig. 6).

In conclusion, our investigation has successfully isolated *L. mesenteroides* from local kefir, identifying this bacterium as a potential probiotic strain. Live *L. mesenteroides* bacteria exhibit a significant capability to upregulate both PD-L1 gene and protein expression in colon cancer cell lines. This effect may be attributed to the activation of peptidoglycan, a component known to activateTLR2 and NOD2, along with the activation of GPCRs, specifically GPR41/42 and GPR43, which act as sensors for SCFAs produced by this bacterium. However, the primary factor responsible for inducing PD-L1 expression requires further investigation. Moreover, our study demonstrates that live *L. mesenteroides* induces Jurkat T cells to secrete IFN-γ and IL-2, signifying its capability to activate T cells and bolster their role in eliminating cancer cells. Additionally, we observed that live *L. mesenteroides* bacteria effectively restore the activity and viability of Jurkat T cells, surpassing control conditions. To further elucidate this observation, an in vivo study is warranted to identify the primary immune cells activated and the specific pathways involved in reducing cancer. These findings underscore the potential of this probiotic to modulate the immune response against cancer. The cumulative evidence from our study suggests that live *L. mesenteroides*, derived from kefir, emerges as a promising adjuvant in PD-L1 immunotherapy blockade. The profound impact of probiotics on immune therapy is highlighted by their ability to modulate immune responses, influence immune checkpoint gene and protein expression, potentially enhancing the efficacy of immune checkpoint inhibitors in cancer treatment. It is essential to note that this investigation serves as a preliminary study in cell culture settings, paving the way for more in-depth examinations in future research endeavors.

### Electronic supplementary material

Below is the link to the electronic supplementary material.

Supplementary Material 1

## Data Availability

The entirety of the data produced or examined during this study is incorporated within the published article.
