# Supplementary material for: Upregulation of Immune checkpoint PD-L1 in Colon cancer cell lines and activation of T cells by Leuconostoc mesenteroides
Source: World J Microbiol Biotechnol. 2024 May 17;40(7):204. doi: 10.1007/s11274-024-04018-7 (PMC11098917; doi:10.1007/s11274-024-04018-7)
Supplement: Supplementary file 1 — Supplementary Material 1 [file 11274_2024_4018_MOESM1_ESM.docx]

**World Journal of Microbiology and Biotechnology**

**Upregulation of Immune Checkpoint PD-L1 in Colon Cancer Cell Lines and activation of T cells by *Leuconostoc mesenteroides***

**Safaa ALTVES^1,2^,** **Ebru GUCLU^3^, Esra YETISGIN^4^, Kivanc BILECEN^4^ and Hasibe VURAL^1^**

^1^Department of Medical Biology, Faculty of Medicine, Necmettin Erbakan University, Konya, Turkey

^2^Science and technology research and application center (BITAM), Necmettin Erbakan university, Konya, Turkey

^3^Department of Basic Science and Health, Hemp Research Institute, Yozgat Bozok University, Yozgat, Turkey

^4^Department of Molecular Biology & Genetics, Faculty of Agriculture and Natural Sciences, Konya Food and Agriculture University

Corresponding author: [safaa.e.t@gmail.com](mailto:safaa.e.t@gmail.com)

**Supplementary data**

**Table 1: primers that used in this research**

| **Gene** | **Forward primer (5'->3')** | **Reverse primer (3'->5')** | **Amplicon** |
| --- | --- | --- | --- |
| **ACTB** | AGCACGGCATCGTCACCAACT | TGGCTGGGGTGTTGAAGGTCT | 179 |
| **ATG16L1** | CGCAGCAAAGTCTGCATAAAAG | CTCTGATCGAATGTCCCAGAAA | 126 |
| **ATG5** | AGCAACTCTGGATGGGATTG | AGGTCTTTCAGTCGTTGTCTG | 140 |
| **ATG7** | GGATGGTGAACCTCAGTGAAT | AGTAGGAACCAATCTCCAACAC | 98 |
| **BCL-2** | GTGGATGACTGAGTACCTGAAC | AGACAGCCAGGAGAAGAATCAA | 125 |
| **BECN1** | GCAGCGGATAAGCTGAAGA | CGACCCAGCCTGAAGTTATT | 99 |
| **CART9** | AGGAAGATGCAGAAAGGATGG | CCTCAGTGTCGGTGTTGTC | 79 |
| **CASP-3** | GAGCCATGGTGAAGAAGGAATA | TCAATGCCACAGTCCAGTTC | 162 |
| **CASP-7** | CGAAACGGAACAGACAAAGATG | TTAAGAGGATGCAGGCGAAG | 169 |
| **CASP-8** | GCCCAAACTTCACAGCATTAG | GTGGTCCATGAGTTGGTAGATT | 160 |
| **CYCS** | GGAGAGGATACACTGATGGAGTA | GCTGCCCTTCTTCCTTCTT | 102 |
| **ERK1** | GCTCCTGACGGAGTATGTG | CAGATGTCGATGGACTTGGTATAG | 91 |
| **ERK2** | GTACAGGACCTCATGGAAACAG | CCTCTGAGGATCTGGTAGAGAA | 97 |
| **GAPDH** | GTCAACGGATTTGGTCGTATTG | TGTAGTTGAGGTCAATGAAGGG | 106 |
| **GPCR41/42** | GGGCTATATCTGCGGTGAAA | GGAGGAGGAGAAGTAGTAGACA | 103 |
| **GPCR43** | CCCTGCTCTTCTATTTCTCTTCTT | CTGCTGTGTCTTTGCCCTCT | 114 |
| **MYD88** | CTGTGTCTGGTCTATTGCTAGTG | TTCCTTGCTCTGCAGGTAATC | 115 |
| **NFKB1** | CACCCTGACCTTGCCTATTT | AGCTGCTTGGCGGATTAG | 87 |
| **NOD 1** | GACAACAACAATCTCAACGACTAC | ACCGTCAGTGATCTGGTTTAC | 99 |
| **NOD 2** | CTGTCTTCTCATGGATGGTGTC | TGCAGAATCAGCAGGTACATATC | 100 |
| **PD-L1** | GCTGAATTGGTCATCCCAGAA | CAGTGCTACACCAAGGCATAA | 102 |
| **SQSTM1** | ATTGAGTCCCTCTCCCAGAT | CGCTCCGATGTCATAGTTCTT | 96 |
| **STK4** | KATAAGAGACCGGCCAGATT | GCGTCCACATTGCTGCATTATAG | 102 |
| **TAZ** | CATATCATTCGAGGGAGCAGAG | CCTGTATCCATCTCATCCCACATT | 109 |
| **TIRAP** | GCGCTGGAGCAAAGACTAT | CAGTGCTGCCTTCCAAGTA | 96 |
| **TLR2** | GGGCTCACAGAAGCTGTAAAA | TCTGTAGGTCACTGTTGCTAATG | 76 |
| **YAP 1** | ATGATGAACTCGGCTCTCAGG | GAGGTGGTCTTGTTCTTATGGT | 101 |


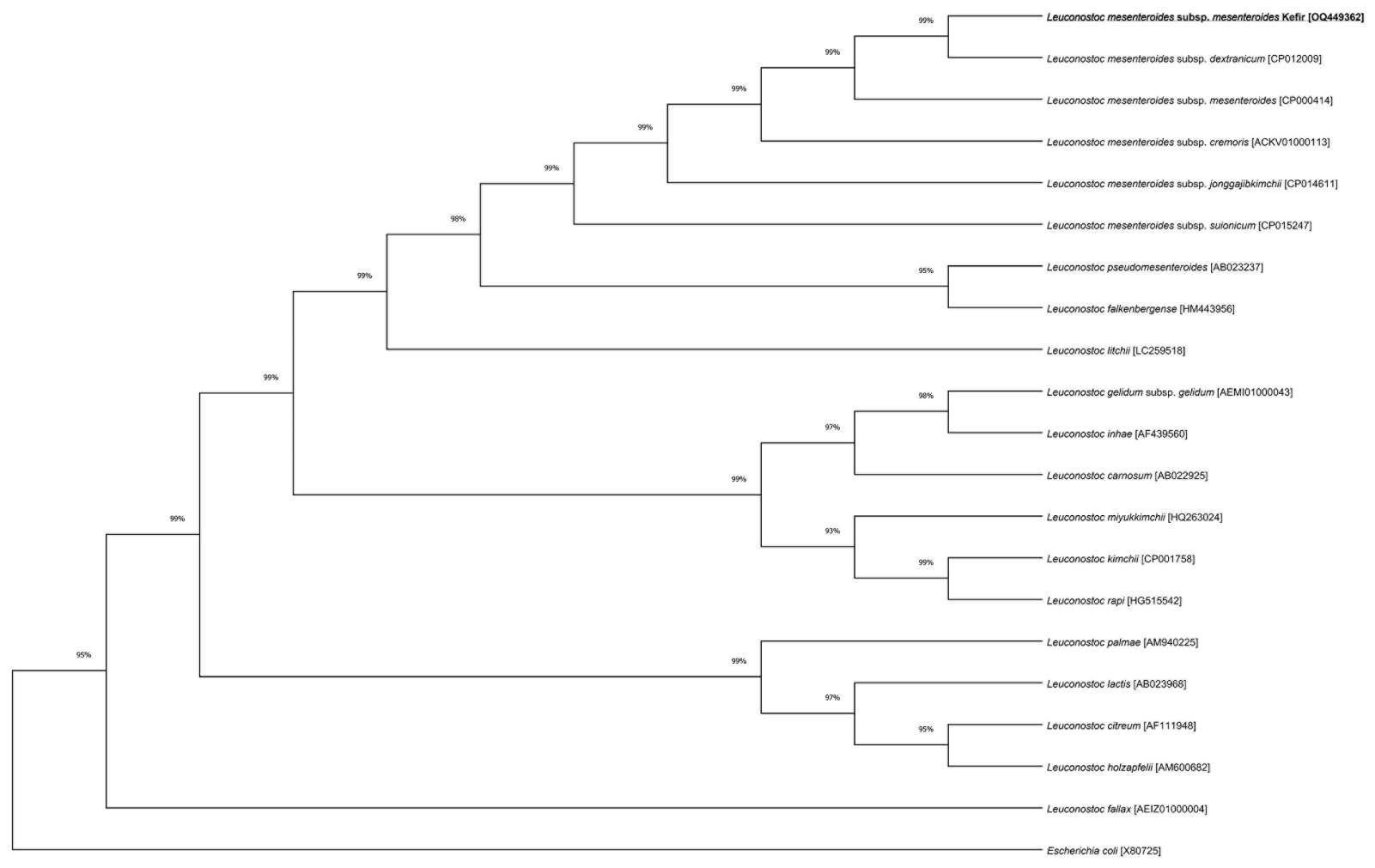


**Figure 1S Phylogenetic tree of *L. mesenteroides* subsp. mesenteroides Kefir.**

This tree was generated based on 16S rRNA gene sequence of the strain. The evolutionary distances were computed as in Ciplak et al. (2023). The tree was constructed on 21 nucleotide sequences, and the final dataset had 1494 places. *Escherichia coli* ATCC-11775 was put as an outgroup

**Reference**

Ciplak, E S, Bilecen K, Akoglu K G, and Guchan N S. (2023) Use of bacterial binder in repair mortar for micro-crack remediation. Appl Microbiol Biotechnol 107, 3113–3127. doi.org/10.1007/s00253-023-12507-2 f
